# Dr. John Howard Ripley

**Published:** 1896-03

**Authors:** 


DR. JOHN HOWARD RIPLEY, of New York, died at Norma, Fla., February 14, 1896, aged 58 years. Dr. Ripley was born in South Coventry, Conn., May 16, 1837. He came to New York in 1861 and entered the medical department of the University of the City of New York, but before finishing his course he entered the army, and remained in the service until the close of the war, when he returned to New York and was graduated in the class of 1866. He began practice at once and soon took a high place in the profession. He became an acknowledged consultant in general practice, his fame extending beyond New York, and he was frequently called to neighboring cities.

Some time ago he was called to Hartford in consultation, and on the journey was attacked with the grip. He returned to New York quite ill, but with that devotion to his work for which he was well known, he refused to rest, and continued to perform the duties of his extensive private practice and his hospital service. He was finally prevailed upon by his life-long friend, Dr. George F. Shrady, to quit work and go south for rest and recuperation, but his illness had already made such inroads that the more genial climate could not restore his health. Dr. Ripley is survived by a widow, one son and two daughters. His remains were brought to New York for interment.

